# Transcriptional regulation of copper metabolism genes in the liver of fetal and neonatal control and iron-deficient rats

**DOI:** 10.1007/s10534-014-9802-z

**Published:** 2014-10-28

**Authors:** Malgorzata Lenartowicz, Christine Kennedy, Helen Hayes, Harry J. McArdle

**Affiliations:** 1 Rowett Institute of Nutrition and Health, University of Aberdeen, Greenburn Road, Bucksburn, Aberdeen, AB21 9SB UK; 2 Department of Genetics and Evolution, Institute of Zoology, Jagiellonian University, Gronostajowa 9, 30-387 Kraków, Poland

**Keywords:** Copper-iron interactions, Perinatal development, Metallochaperones, ATP7A, ATP7B, Copper metabolism, Hooded Lister rats

## Abstract

Copper and iron metabolism have been known to interact for many years. We have previously shown, during pregnancy, that copper levels in the maternal liver rise as a consequence of iron deficiency, but that levels in the fetal liver decrease. In this paper, we measure expression of genes involved in copper metabolism in fetal and postnatal liver, to test whether alterations can explain this observation. Additionally, we study the extent to which gene expression changes in the latter stages of pregnancy and in the perinatal period. *Ctr1* expression levels dropped to term, rising again thereafter. There was no difference in gene expression between control and iron deficient animals. *Atox1* expression remained approximately stable until term, and then there was a rise to a maximum at about Day 8. *Atp7a* expression levels remained constant, except for a brief drop at term. *Atp7b* levels, in contrast, decreased from a maximum early in gestation to low levels in the term and post-natal livers. Ceruloplasmin expression appeared to be diametrically opposite to *Atp7b*. The other two metallochaperones showed the same pattern of expression as *Atox1*, with a decrease to term, a rise at Day 1, or a rise after birth followed by a brief decrease at about Day 3. None of the genes were significantly affected by iron deficiency, suggesting that changes in expression cannot explain the altered copper levels in the fetal and neonatal liver.

## Introduction

Copper is a micronutrient, essential for normal growth and development. It is a cofactor for enzymes that catalyze reactions used in fundamental metabolic processes, including respiratory oxidation, neurotransmitter synthesis, iron uptake regulation, and connective tissue formation (Lutsenko et al. 2007). However, an excess of copper has the potential to be toxic, as the ions are potent generators of free radicals, which could lead to oxidative damage of proteins, lipids and nucleic acids (Lutsenko et al. 2007; Van den Berghe and Klomp 2010; Velthuis et al. 2009). Consequently, organisms have developed complex and genetically regulated control mechanisms for maintaining the balance between essential and toxic copper levels.

In mammals, copper is absorbed mainly in the stomach and duodenum and is transported in plasma to the liver (Van den Berghe and Klomp 2010), the main organ responsible for copper homeostasis. The copper is stored primarily as a copper-metallothionein complex (Bauerly et al. 2005; Czachor et al. 2002; Davis and Cousins 2000) and is excreted through bile (Huster et al. 2006; Wijmenga and Klomp 2004).

**Fig. 1:**
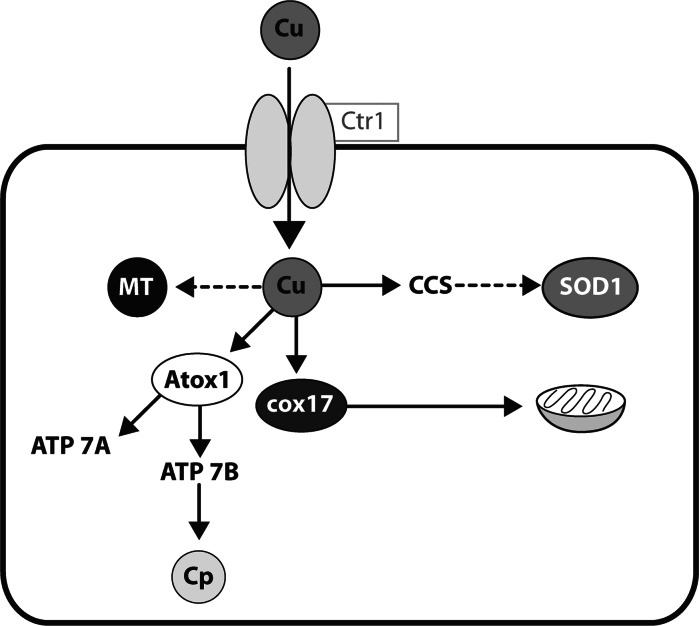
The model used to study gene expression in this paper. Copper is taken up across the cell membrane, binds to the relevant chaperone and is incorporated into the target protein. More information is given in the text in the relevant section

Copper uptake by hepatocytes is mediated by the high affinity copper transporter 1 (CTR1) (Kuo et al. 2001, 2006). In the cell the copper is bound to metallochaperones, and transported to the different compartments of the cell (O’Halloran and Cizewski Culotta 2000). Different chaperones deliver the copper to different enzymes. The copper chaperone of superoxide dismutase (CCS) partitions copper for SOD synthesis (Wong et al. 2000; Bertinato et al. 2003; Prohaska et al. 2003). Copper ions bound to COX 17 chaperone are transported to mitochondria where they are bound to the subunits of the cytochrome-c oxidase (Nevitt et al. 2012; Kako et al. 2004; Punter and Glerum 2003). ATOX1 transport copper to the trans-Golgi network where it is incorporated into the two Cu-transporting ATPases, ATP7A and ATP7B proteins (Barry et al. 2010; Pufahl et al. 1997; Lutsenko et al. 2007, 2008). In hepatocytes, ATP7B delivers copper to ceruloplasmin, a major form of copper in serum. It is also the pathway of excretion of copper into the bile (Huster et al. 2006; La Fontaine et al. 2001; Wijmenga and Klomp 2004). ATP7A is not expressed in the adult liver, but in other tissues is involved in copper excretion and absorption. These data are summarized in Fig. 1.

Metabolic interactions between the two essential elements, copper and iron, have been known for many years (McArdle 1992; McArdle et al. 2008; Gambling et al. 2011). In the early part of the last century it was shown that copper could facilitate hemoglobin formation in anemic rats (Hart et al. 1928). More recently, it has been demonstrated that iron metabolism is critically dependent on ferroxidases, most of which are copper enzymes. How iron alters copper metabolism is not so well understood. We have previously shown that iron deficiency results in accumulation of copper in the maternal, but not fetal liver. How this occurs is not clear but is presumably related to alterations in gene expression in one of the major organs involved in iron and copper transfer from mother to fetus. In this paper, therefore, we examine the effect of iron deficiency on expression of the genes of copper metabolism, with a view to identifying the changes that could explain alterations in copper levels in the maternal and fetal livers.

## Materials and methods

### Animals and diets

#### Ethical approval

All animal experiments were performed in the Bioresources Unit of the Rowett Institute of Nutrition and Health, under licence from the United Kingdom Home Office in accordance with the UK Animals (Scientific Procedures) Act, 1986. The study was approved by the UK Home Office (Project Licence PPL 60/3606) and the Rowett Institute of Nutrition and Health Ethics Review Committee (approval ID SA09/03E).

#### Experimental diets

The experimental diets were based on a dried egg albumin diet and conformed to American Institute of Nutrition guidelines for laboratory animals (Williams and Mills 1970). FeSO_4_ was added to achieve levels of added iron of 50 (control diet) and 7.5 (iron deficient diet) mg kg^−1^. Dietary ingredients were purchased from Mayjex Ltd (Chalfont-St Peter, UK), BDH Chemicals (Poole, UK) or Sigma (Poole, UK). Diet formulations have all previously been used (Gambling et al. 2003a).

#### Experimental animals and tissue collection

Experiments were performed using weanling female rats of the Rowett Hooded Lister strain. Animals were housed in cages under constant conditions (temperature, humidity, 12L:12D illumination photoperiod). Female weanling rats were fed control diet for 2 weeks, before being randomised into two groups. The first group remained on the control diet throughout the experiment, including pregnancy, whilst the scond were given the iron deficient diet for 4 weeks prior to mating. The rats were mated with males of the same strain. Mating was confirmed by detection of a vaginal plug, and this day was denoted as Day 0.5. The female rats were maintained on the same diet throughout pregnancy.

At times stated in the results, 8 rats from each group were killed as described below. At parturition, blood and tissue samples were taken from 8 dams and their litters, from birth the control and iron deficient groups. Following parturition all remaining dams fed on control diet during pregnancy continued on this diet. At the same time dams fed on the iron deficient diet during pregnancy were split into two groups, half remained on the iron deficient diet, whilst the diet for the remaining dams was changed to the control diet. Within 24 h of birth all litters were culled to 8 pups, with male pups being kept preferentially. Over the next 10 days, there were 4 time points at which 8 dams and their litters from each of the now 3 groups, were killed and blood and tissue samples taken. These time points were 1, 3, 7 and 10 days after birth.

Dams were anesthetized with isoflurane, and maternal blood samples collected from the heart. The dams were then killed by cervical dislocation. Tissue samples were also taken from the dam. The fetuses or neonates were weighed, and then killed by decapitation. Blood samples were collected from each of the neonates, and pooled to provide a representative litter sample. Organs were weighed and snap frozen in liquid nitrogen before being stored at −80 °C. In order to obtain representative samples, tissues from the 3 pups closest to the litter median, determined by body weight, were pooled and ground. These pooled samples were used for all future analysis.

### RNA isolation

Total RNA was prepared from 50 mg of frozen liver tissue by homogenizing in cold TRI reagent (Helena Biosciences, Sunderland, UK) according to the manufacturer’s instructions. RNA were precipitated in isopropanol and dissolved in DNAse, RNASe free water. Concentration of RNA in samples was measured in a NanoDrop™ 2000c spectrophotometer (Thermo Scientific) OD 260/280 and purify of RNA was estimated using OD 260/230. RNA integrity was determined using Agilent 2100 Bioanalyzer^®^ (Agilent Technologies) according to the manufacturer’s instructions. To avoid contamination of obtained RNA with genomic DNA, 1 ug of total RNA samples were treated with ribonuclease (RNase)-free Dnase 1 Amplification Grade (Invitrogen Ltd, Paisley, UK), before the reaction of reverse transcription. Reverse transcription was performed according to the manual instruction using the Taqman RT Reagent Kit (Applied Biosystem) from 200 ng of DNAse-treated RNA.

### Real time PCR reaction

Real-time quantitative PCR was performed using the 7700 Real Time PCR system (Applied Biosystem) and the primers specific for analysed gene (Table 1). Primers for Atp7a and Atp7b genes were designed, using Primer Express software and were ordered in MWG Eurofins Company, sequence of primers is showed in Table 1. Primers for metallochaperones, *Ctr1*, ceruloplasmin (*Cp*) and ubiquitin genes (Table 1) obtained from Qiagen. Quantification was performed relative ubiquitin control reaction. To confirm amplification specificity, the PCR products from each primer pair were subjected to melting curve analysis and subsequent agarose gel electrophoresis. The relative expression of each gene was calculated as 2^−ΔΔCT^.

**Table 1:** Primer list used for real-time quantitative RT-PCR analysis

Gene	Sequence/cat. no
MWG Eurofins primers made to order	Sequence
Atp7a forward	5′-aggcaaatttccagtggatg-3′
Atp7a reverse	5′-atgaggagcgatccattctg-3′
Atp7b forward	5′-acatgctaatccccagcagt-3′
Atp7b reverse	5′-gatgagcacatccatgttgg-3′
Quantitect primer assays	Cat. no
Atox1	QT01081696
Ctr1 (Slc31a1)	QT00175161
Cox17	QT00183827
CCS	QT00181286
Cp1	QT00495075
UBQc	QT00372596

### Statistical analysis

All data are expressed as mean ± SEM. Comparison of the relative expression level were made by one-way ANOVA followed by the NIR Fisher post hoc test. Student *t* test was used when comparing data between control and Fe-deficient rats. Values of *p* < 0.05 were considered statistically significant.

## Results

**Fig. 2:**
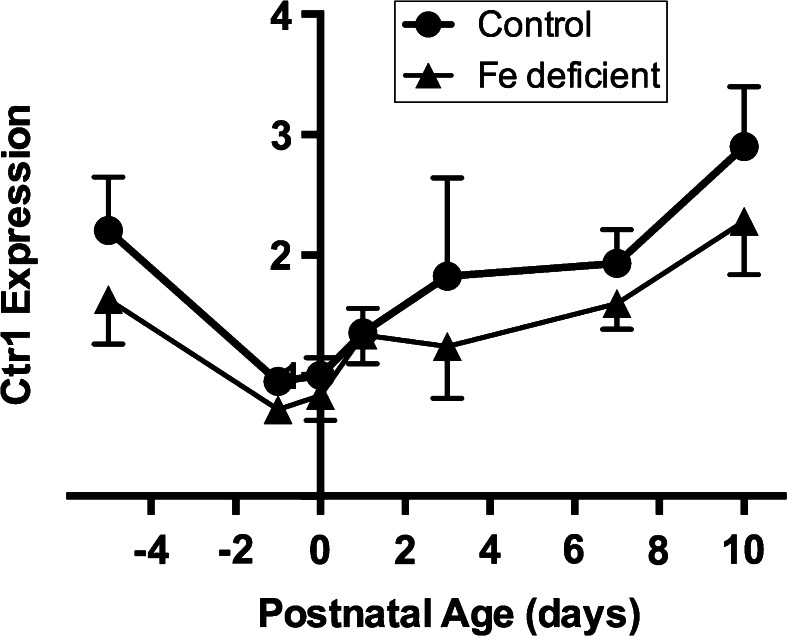
Developmental changes in the expression level of the *Ctr1* gene in the liver of the control and Fe deficient fetus and neonates. The mRNA level are shown relative to ubiquitin. Values with different letters differ significantly (*p* < 0.05). Data are expressed as mean ± SEM

**Fig. 3:**
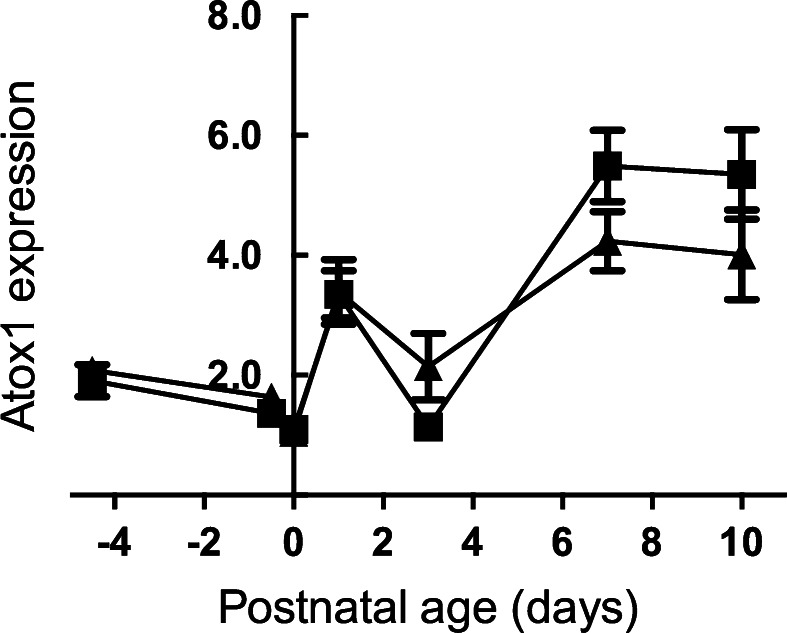
Expression pattern of the *ATOX1* gene in the liver of the control and Fe deficient rats between 17.5 and 21.5 of gestation and Day 0 to Day 10 of the postnatal life. The mRNA level is shown relative to ubiquitin. Values with different letters differ significantly. Data are expressed as mean ± SEM

In the liver of both normal and iron deficient rats expression of the *Ctr1* gene decreased from 17.5 to the 21.5 days gestation, then increased postnatally. There was no significant difference in expression between control and Fe deficient livers, though there was an apparent trend (*p* = 0.07) (Fig. 2). After entry to the hepatocytes, copper is bound to chaperone proteins. We measured mRNA levels of *Atox1* in control and iron deficient fetal livers. There were no significant differences between the two groups at any stage of gestation or postnatal life (Fig. 3). As with *Ctr1*, levels decreased to term. After birth, levels rose in both control and Fe deficient pups. However, there was a decrease at Day 3. Currently, we do not have an explanation for this observation.

**Fig. 4:**
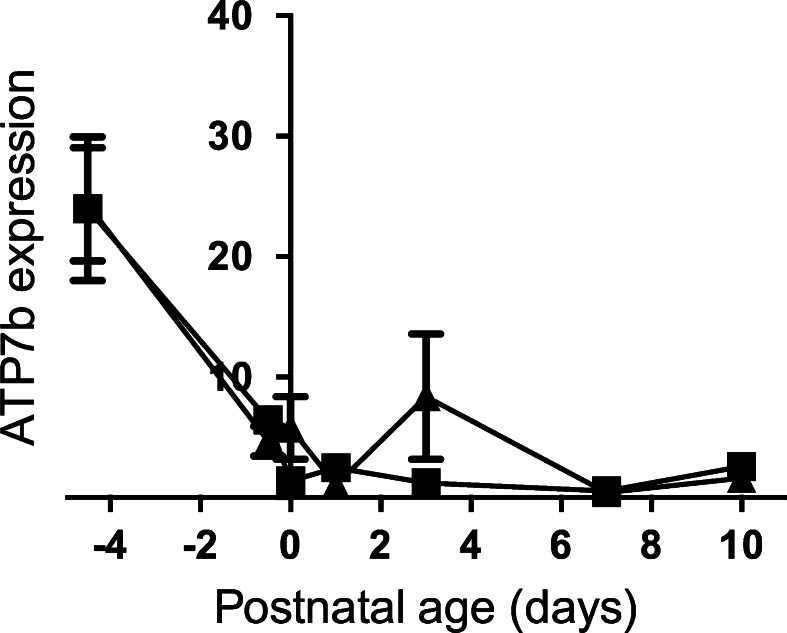
Developmental changes in the expression level of the *Atp7b* gene in the liver of the control and Fe deficient fetus and neonates. The mRNA level is shown relative to ubiquitin. Values with different letters differ significantly (*p* < 0.05)

**Fig. 5:**
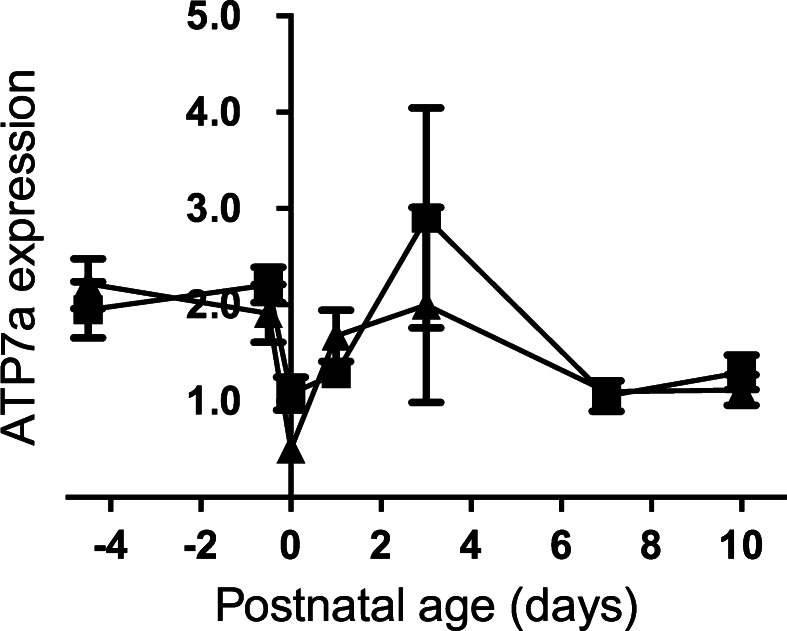
The *Atp7a* mRNA profile of the liver of the control and Fe deficient fetus and neonates. The mRNA level is corrected relative to ubiquitin. Values with different letters differ significantly (*p* < 0.05)

ATOX1 protein delivers the copper ions to the Golgi apparatus, where they bind to the Cu-transporting ATPases, ATP7A and ATP7B. Both *Atp7a* and *7b* genes were expressed in the fetal liver. Levels dropped to term, and did not change subsequently for *Atp7b* (Fig. 4). In contrast, *Atp7a* expression rose briefly at Day 3 (see above for *Atox1*) thereafter returning to levels seen at Day 0 (Fig. 5). In adult liver, ATP7A expression is negligible. However, in the neonates, expression levels were significantly higher than *Atp7b*, implying that the former may play a significant role in hepatic copper metabolism in the neonate.

**Fig. 6:**
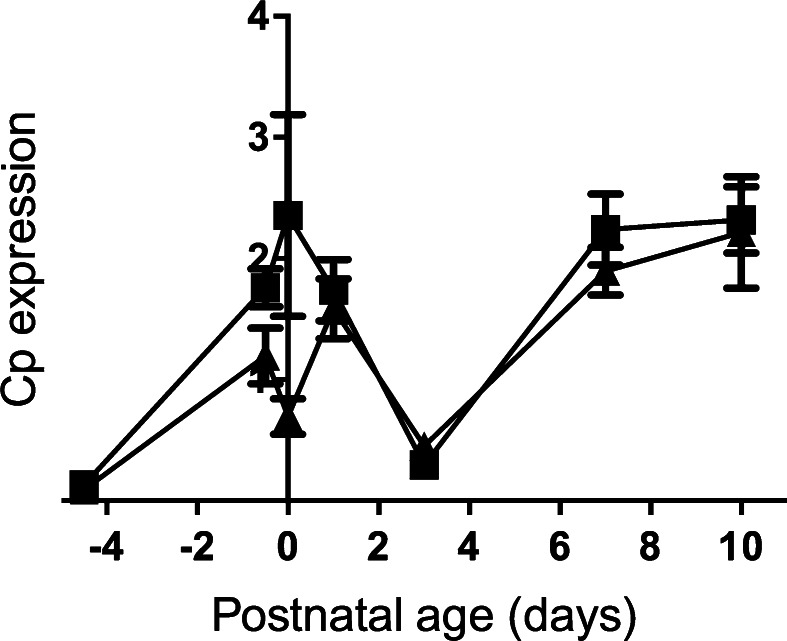
Ceruloplasmin mRNA level in the liver through developmental stages of the control and Fe deficient fetus and neonates. The mRNA level is shown relative to ubiquitin. Values with different letters differ significantly (*p* < 0.05). Data are expressed as mean ± SEM

The ATP7B Cu-ATPase delivers copper to ceruloplasmin, so we determined expression levels of *Cp* in the fetal and neonatal liver. Before birth, expression increased in the fetal liver, dropped to their lowest levels at Day 3 and thereafter increased (Fig. 6). There was no difference in mRNA levels between control and Fe-deficient animals. Further, expression levels of *Cp* were not correlated with expression of *Atp7b* (data not shown).

**Fig. 7:**
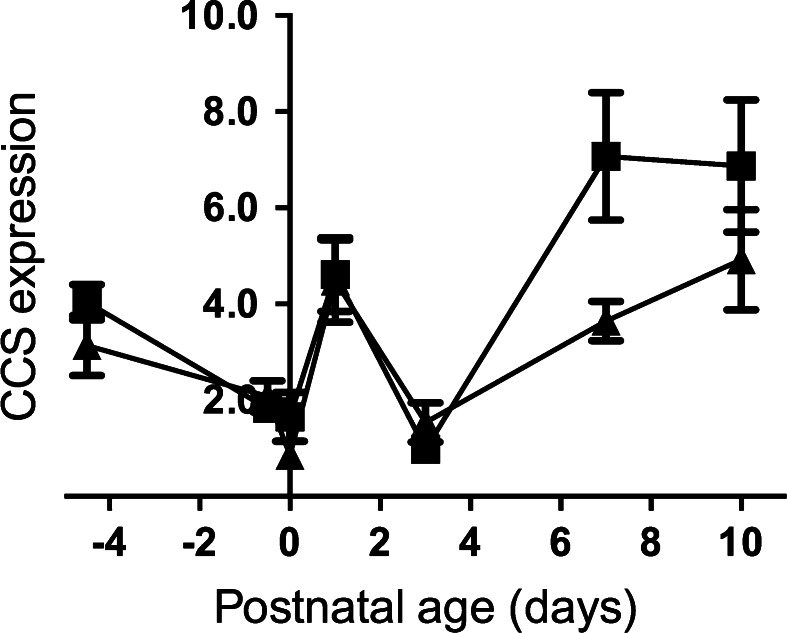
Relative expression of the *CCS* gene using ubiquitin as the endogenous normalization control in the liver of the control and Fe deficient fetus and neonates. Values with different letters differ significantly (*p* < 0.05). Data are expressed as mean ± SEM

Copper bound to CCS is used for SOD1 synthesis. In both control and iron deficient animals, *CCS* levels dropped during pregnancy, rose in the immediate post-natal period, then fell again at Day 3 (Fig. 7). The increase in expression after Day 3 was significantly greater in controls than in animals from iron deficient dams.

**Fig. 8:**
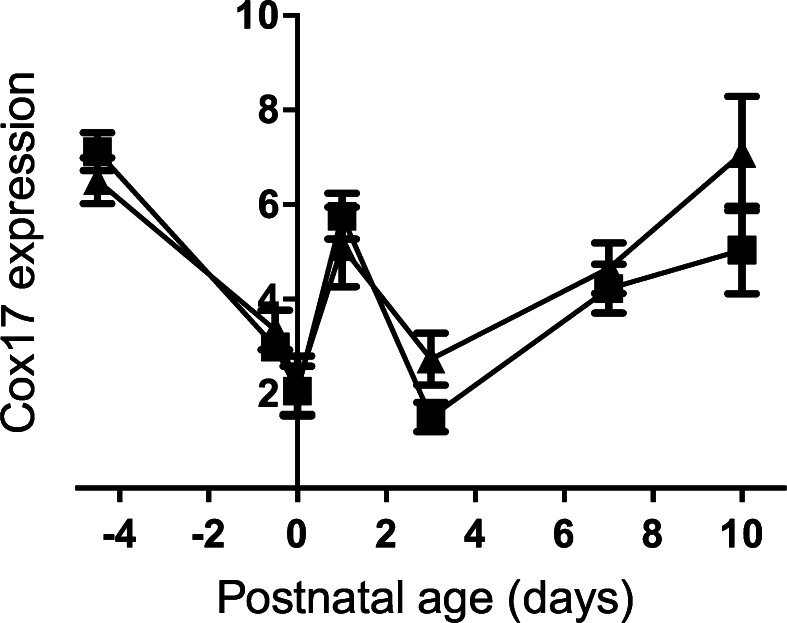
Developmental changes in the expression level of the *Cox17* gene in the liver of the control and Fe deficient fetus and neonates. The mRNA level is shown relative to ubiquitin. Values with different letters differ significantly (*p* < 0.05). Data are expressed as mean ± SEM

Finally, *Cox17* expression was measured. As with the other chaperones, there was a drop followed by a rise at birth, thereafter falling back at Day 3 (Fig. 8). From then, there was a rise until the end of the experiment at Day 10 post-natal. The difference between control and Fe-deficient animals was not significant.

## Discussion

Copper and iron are micronutrients essential for normal fetal development. Both are transferred from mother to fetus across the placenta (Gambling et al. 2003b; Hardman et al. 2007; McArdle et al. 2003). The metabolisms of the two elements have been known to interact for many years (reviewed in Collins et al. 2010; McArdle 1992; Gambling et al. 2011). Iron release from cells is dependent on copper oxidases; hephaestin in the gut, ceruloplasmin in the liver and zyklopen in the placenta. Copper deficiency, therefore, results in accumulation of iron in the liver, and in the kidney and brain. The effect of iron deficiency on copper metabolism is not so well studied. We have shown, as have others (Sherman and Moran 1984; Sherman and Tissue 1981) that, during pregnancy, copper levels in the fetal liver decrease in response to maternal iron deficiency, in contrast to the maternal liver, where iron deficiency results in an increase in copper levels (Gambling et al. 2004). How this occurs is not known. In this paper, we test the hypothesis that alterations in expression levels of the genes involved in copper metabolism are involved in the mechanism. Further, we also examine how gene expression changes as development proceeds through late gestation and the early neonatal period.

Terao and Owen (1977) reported that in rats, the copper concentration in the fetal liver was highest at day 18 of pregnancy and decreased to day 21. After birth, copper levels significantly increased up to postnatal day 7. From then, levels remained high until about the second week of life, thereafter decreasing to adult levels by six weeks of age (Terao and Owen 1977). In mammalian cells, copper import is primarily mediated by CTR1 protein (Ansede et al. 2009; Kim et al. 2009; Lee et al. 2002; Nose et al. 2006; Van den Berghe and Klomp 2010). Kuo et al. (2006) showed that in mice and rats CTR1 expression is regulated by copper status (Bauerly et al. 2005; Kuo et al. 2006). In the present study we found that *Ctr1* gene expression matched these patterns. We also found that in neonatal rats after birth *Ctr1* expression in the liver is rather low and gradually increased. There was no apparent effect of iron deficiency on expression of the *Ctr1* gene at any stage in gestation.

Following uptake across the cell membrane, copper is bound to one of a class of proteins called metallochaperones. Several have been identified, each of which will deliver copper from the membrane to a particular target protein. These targets are shown in Fig. 1.

Anti-oxidant 1 protein (ATOX1) (Klomp et al. 1997), transports Cu to ATP7A and ATP7B, the Cu-transport ATPases (Lutsenko et al. 2007, 2008; Van den Berghe and Klomp 2010). In adult liver, ATP7A expression is extremely low (Lenartowicz et al. 2010). However, in the fetus and neonate, both ATPases are expressed. Interestingly, expression levels of *Atp7a*, but not *Atp7b*, and *Atox1* are inversely proportional. Why this should be is not clear, but it could be related to either regulation of *Atp7a* by ATOX1, or possibly to the transcription factor function of ATOX1. Itoh and co-workers showed that copper promotes transport of ATOX1 protein to the nucleus where it is bound to the promoter of regulated genes resulting in up-regulation of Cycline D1, (*Ccnd 1*), and extracellular SOD (SOD3) genes (Itoh et al. 2008, 2009).

In hepatocytes, ATP7B protein is localized in the trans-Golgi network (Schaefer et al. 1999; Bingham et al. 1996) where it is involved in the process of Cu binding to apo-ceruloplasmin (Lutsenko et al. 2007; Terada et al. 1998). It also transfers copper from the hepatocyte to the bile in the case of copper excess (Bauerly et al. 2005; Roelofsen et al. 2000; Huster et al. 2006; Wijmenga and Klomp 2004). In the fetus, the bile duct is not patent, and copper accumulates in the liver (Guo et al. 2005; Lutsenko et al. 2003; La Fontaine and Mercer 2007; Mercer et al. 2003; Schaefer et al. 1999), although it may also be that the copper is accumulated in order to provide a store for the perinatal period.

This is the first study to compare *Atp7b* and *Atp7a* expression levels in the liver of fetal and neonatal animals. Previous work from our laboratories showed developmental regulation of *Atp7a* expression in the liver of neonatal mice (Lenartowicz et al. 2010, 2011) but little is known about expression pattern of *Atp7b* in the liver. Here, we show that in the liver of the neonatal rats *Atp7a* expression dropped in the perinatal period, but then stabilised, while *Atp7b* expression remained low. The data suggest that, in the liver of very young animals, *Atp7a*/ATP7A plays the dominant role in regulation of copper metabolism. By adulthood, this is reversed, and *Atp7b*/ATP7B is the only transporter. Alternatively, the data could reflect the relatively important role the fetal and neonatal liver plays in haematopoiesis, and the high concentration of haematopoietic cells in the liver.

Ceruloplasmin is a ferroxidase, containing 6 Cu atoms per molecule protein, and is required for oxidation of Fe2+ to Fe3+ and thus for iron efflux from the cells via ferroportin. Ceruloplasmin is also the major plasma copper binding protein (Tran et al. 2002; Hellman and Gitlin 2002; Broderius et al. 2010; Prohaska 2011). Incorporation of copper to the ceruloplasmin is mediated by ATP7B protein (Terada et al. 1998). During development, ceruloplasmin expression starts very early (Fleming and Gitlin 1990; Lee et al. 1993) and mRNA in the liver was detected at E15. Expression increases between 17th and 20th day of gestation and drops just before delivery (Fleming and Gitlin 1990). Such findings closely overlap with our own results. Terao and Owen (1977) reported that ceruloplasmin protein levels in the liver of rat pups increase sharply in the first 24 h after birth, which also fits with our data. Ceruloplasmin gene expression unexpectedly significantly decreased at Day 3. In rats in the 3th day postpartum copper levels in the mother’s milk are highest and drop thereafter (Terao and Owen 1977). We speculate that decreasing ceruloplasmin synthesis at this stage may protect the neonates against excess copper. As copper in the milk decreases thereafter, *Cp* levels rise again. Both in vitro experiments on isolated hepatocytes (McArdle et al. 1990) and in vivo studies on mice and rats indicated that copper deficiency have no impact on ceruloplasmin mRNA in the liver (Prohaska 2011; Mercer et al. 1991; Mostad and Prohaska 2011) and our current data also show no difference between control and Fe deficient livers.

Copper, zinc superoxide dismutase (SOD1) protects cells against reactive oxygen species that are generated during the processes of oxidative metabolism. Activity of SOD1 is closely dependent on its copper chaperone, CCS, which is necessary for insertion of Cu ions to apo-SOD1 (Wong et al. 2000). Copper supplementation results in decreasing of CCS and SOD1 mRNA level both in the liver and peripheral mononuclear blood cells (Suazo et al. 2008; Han et al. 2009). Here, we found a significant decrease in CCS expression, which may also be caused by the increase in Cu levels. It is known that in pups born from Fe deficient mothers copper concentration in the liver is significantly increased (Sherman and Moran 1984; Sherman and Tissue 1981). In both investigated group of rats *CCS* mRNA level rapidly and significantly increased during first 24 h of postnatal life.

Cytochrome c oxidase is a terminal complex in the respiratory chain (Oswald et al. 2009; Voronova et al. 2007). Uptake, transport and delivery of the copper ions to the enzyme are complicated processes; requiring activity at least six copper chaperones (Voronova et al. 2007). One of these is COX 17, a small hydrophobic protein which contains copper binding domains, binds and transports copper ions in the cytoplasm and also participates in shuttling Cu ions through the mitochondrial membrane to the intermembrane mitochondrial space (Oswald et al. 2009; Voronova et al. 2007; Kako et al. 2004; Punter and Glerum 2003). In rats *Cox 17* expression in the brain and heart was detected in prenatal life and it was shown that the expression profile was age- and tissue-dependent and changes during development (Kako et al. 2000). In the present study we analysed expression of the *Cox 17* in the liver of the fetus and neonatal rats and found similar results. In the iron deficient animals after birth, *Cox17* expression was higher than control. This may be related to different nutritional profiles in the iron-deficinet dams, but may also be a consequence of reduced mitochondrial function in the iron deficient animals.

In summary, the data presented show that, while there are marked changes in the expression of the genes of copper metabolism while development proceeds, the consequences of iron deficiency are limited and cannot explain the increasing copper levels in the iron deficient liver.
